# Somatic copy number alteration predicts clinical benefit of lung adenocarcinoma patients treated with cytokine-induced killer plus chemotherapy

**DOI:** 10.1038/s41417-021-00422-5

**Published:** 2022-01-12

**Authors:** Fan Kou, Lei Wu, Ye Zhu, Baihui Li, Ziqi Huang, Xiubao Ren, Lili Yang

**Affiliations:** 1 Department of Immunology, Tianjin Medical University Cancer Institute and Hospital, Tianjin, China; 2 National Clinical Research Center for Cancer, Tianjin, China; 3 Key Laboratory of Cancer Immunology and Biotherapy, Tianjin, China; 4 Key Laboratory of Cancer Prevention and Therapy, Tianjin, China; 5 Tianjin’s Clinical Research Center for Cancer, Tianjin, China; 6 Department of Breast Cancer, Tianjin Medical University Cancer Institute and Hospital, Tianjin, China; 7 Department of Biotherapy, Tianjin Medical University Cancer Institute and Hospital, Tianjin, China

**Keywords:** Biomarkers, Non-small-cell lung cancer

## Abstract

Somatic copy number alterations (SCNA), which are widespread in cancer, can predict the efficacy of immune checkpoint inhibitors in non-small-cell lung cancer (NSCLC). However, the usefulness of SCNA for predicting the survival of patients treated with cytokine-induced killer (CIK) cells or chemotherapy (CT) is unknown. This study aimed to explore the correlation between SCNA and clinical outcome in NSCLC patients treated with CIK + CT or CT alone. We performed whole-exome sequencing on 45 NSCLC patients treated with CIK + CT, as well as 305 NSCLC patients treated with CT alone, from The Cancer Genome Atlas, which showed SCNA had a superiority in predicting the progression-free survival (PFS) over tumor mutation burden (TMB) and SCNA + TMB in NSCLC patients treated with CIK + CT, especially in lung adenocarcinoma, while SCNA could not predict the efficacy of CT alone. Additionally, we investigated the association between SCNA and immune cell infiltration by RNA sequencing and immunohistochemistry. The results revealed that SCNA was negatively associated with the expression of dendritic cells. Collectively, this study revealed a negative correlation between SCNA and response to CIK + CT and showed that SCNA is a predictive indicator in LUAD patients treated with CIK + CT.

## Introduction

Non-small cell lung cancer (NSCLC), comprising lung adenocarcinoma (LUAD) and lung squamous cell carcinoma (LUSC), is a leading cause of malignancy-related mortality [1]. Despite the increasing use of lung cancer screening and improvements in treatment, most NSCLC patients are still diagnosed with advanced cancer, and their prognosis remains dismal [2]. Cytokine-induced killer (CIK) cells are a population of cytotoxic T lymphocytes, which are characterized by the CD3 + CD56 + phenotype, and display wide MHC-unrestricted antitumor activity and potential effectiveness against several cancer types [3, 4]. Recent studies have reported that the survival of NSCLC patients with CIK plus chemotherapy (CIK + CT) was significantly higher than patients who received either CIK or CT alone [5, 6]. However, not all NSCLC patients who receive CIK + CT exhibit improved clinical outcomes. Therefore, we sought to investigate the predictors that can identify patients who are likely to benefit from CIK + CT treatment.

Somatic copy number alterations (SCNA), known as aneuploidy, are common in cancer and involve alterations in a large portion of the cancer genome [7, 8]. The high frequency of SCNA during tumorigenesis, progression, and recurrence has been found to be a predictor of the efficacy of immunotherapy in melanoma [8]. Tumor mutational burden (TMB) is the total number of mutations in cancer. A tumor with a higher TMB is more likely to have neoantigens, which induce the immune cells to target tumor cells [9]. TMB is associated with response to immunotherapy [10]. In NSCLC and melanoma, high SCNA was associated with poorer patient survival, and SCNA levels were a better predictor of survival in patients [11, 12]. Davoli et al. found that most of the gene expression signatures with significant downregulation in high SCNA tumors were signatures related to immune cells including CD4 + T cells, CD8 + T cells, macrophages, dendritic cells (DC), and natural killer (NK) cells [8]. As CIK cells are T cells that can kill tumor cells, it remains unclear whether SCNA can distinguish NSCLC patients that may benefit from CIK + CT treatment.

In this study, we performed whole-exome sequencing (WES) on samples from NSCLC patients treated with CIK + CT and explored whether SCNA could function as a biomarker for patients treated with CIK + CT or CT alone. Additionally, we investigated the association between SCNA and immune cell infiltration.

## Methods

### Patients and treatment

A total of 45 NSCLC patients treated with CIK at Tianjin Medical University Cancer Institute and Hospital between 2006 and 2015 were enrolled in this study. Detailed patient data were shown in Table 1. CIK preparation was described in our published studies [13]. Peripheral blood mononuclear cells were collected from patients using a Code Spectra Apheresis System (Caridian BCT, Lakewood, USA). Then, these cells were cultured in a medium containing 50 ng/mL anti-CD3 antibody (e-Bioscience, San Diego, USA), 1000 U/mL interferon-γ (IFN-γ), and 100 U/mL recombinant human interleukin (IL)−1α to induce CIK cells at 37 °C with 5% CO2 for 24 h. Subsequently, 300 U/mL of recombinant human IL-2 was added to the medium, which was regularly replaced with a fresh medium containing IFN-γ- and IL-2 every 5 days. On day 14, the CIK cells were harvested, and the median number of CIK cells was 7.4 × 109. This method produced a significantly higher proportion of the CD3 + CD56 + cellular subset. Patients received 4 cycles of autologous CIK cell infusions on days 15 and 16 (total count of CIK cells ≥1 × 10^10^) with 4 weeks per cycle. Patients received chemotherapy on day 1 or days 1 and 8, and following the CIK infusion on days 15 and 16 at an interval of 1 month. All patients received chemotherapy with the TP regimen (paclitaxel, 135 mg/m^2^, day 1; cisplatin, 80 mg/m^2^, day 1), GP regimen (gemcitabine, 1000 mg/m^2^, days 1 and 8; cisplatin, 80 mg/m^2^, day 1), or NP regimen (navelbine, 25 mg/m^2^, days 1 and 8; cisplatin, 80 mg/m^2^, day 1). This study was approved by the National Medical Products

**Table 1:** Patient Characteristics.

Characteristic	Total (*n* = 45)	LUAD (*n* = 27)	LUSC (*n* = 18)
Gender, *n* (%)			
Male	34 (75.56%)	18 (66.67%)	16 (88.89%)
Female	11 (24.44%)	9 (33.33%)	2 (11.11%)
Age, y, *n* (%)			
<60	25 (55.56%)	15 (55.56%)	10 (55.56%)
≥60	20 (44.44%)	12 (44.44%)	8 (44.44%)
Smoking, *n* (%)			
No	20 (44.44%)	15 (55.56%)	5 (27.78%)
Yes	25 (55.56%)	12 (44.44%)	13 (72.22%)
Tumor stage, *n* (%)			
І	22 (48.89%)	12 (55.56%)	10 (55.56%)
II	11 (24.44%)	7 (25.93%)	4 (22.22%)
III	9 (20.00%)	6 (22.22%)	3 (16.67%)
IV	3 (6.67%)	2 (3.8%)	1 (5.55%)
NSCLC, *n* (%)			
LUAD	27 (60.00%)	27 (100%)	0
LUSC	18 (40.00%)	0	18 (100%)
Progression, *n* (%)			
No	32 (71.11%)	17 (62.96%)	15 (83.33%)
Yes	13 (28.89%)	10 (37.04%)	3 (16.67%)
Survival, *n* (%)			
No	6 (13.33%)	5 (18.52%)	1 (5.56%)
Yes	39 (86.67%)	22 (81.48%)	17 (94.44%)

Administration (China) (2006L01023) and by the Ethics Committee of the Tianjin Medical University Cancer Institute and Hospital (Approval No. E2016055), according to the guidelines of the Declaration of Helsinki.

### Whole exome sequencing and RNA sequencing

A total of 0.6 μg genomic DNA per sample was used as input material for the DNA sample preparation. Exome capture was performed using Agilent SureSelect Human All Exon V6 kit (Agilent Technologies, CA, USA) following the manufacturer’s recommendations. The clustering of the samples was performed on a cBot Cluster Generation System by using a Hiseq PE Cluster Kit (Illumina) according to the manufacturer’s instructions. After cluster generation, the DNA libraries were sequenced on the Illumina Hiseq platform and generated 150 bp paired-end reads. A total of 2 μg RNA per sample was used as input material for the RNA sample preparation. RNA Sequencing libraries were produced using NEBNext® UltraTM RNA Library Prep Kit for Illumina® (NEB, USA) following the manufacturer’s recommendations and index codes were added to attribute sequences to each sample.

### Sequencing data analysis

WES data were mapped to the reference human genome (UCSC hg19) with Burrows-Wheeler Aligner (BWA) software23 to obtain the original mapping results stored in BAM format. And SCNA were determined from ABSOLUTE, described in published studies [8]. SCNA levels were grouped into low and high SCNA based on the median. In The Cancer Genome Atlas (TCGA) cohort, we selected the number of segments as SCNA, as the method was consistent with our analysis. TMB was calculated using the total number of mutations per Mb and similarly classified based on the median. The score of combined SCNA and TMB was measured by logistic regression. SCNA data was shown in Supplementary Table 1. RNA sequencing data was shown in Supplementary Table 2.

For RNA sequencing data, clean paired-end read was aligned to the human reference genome (hg19) with Hisat2 (v2.0.5)26, that was guided by the gene model Ensembl GRCh37.87. HTSeq (v0.11.2) was used to count the read numbers mapped to each gene.

### TIMER database analysis

TIMER is a web resource for analyzing the gene expression of tumor-infiltrating immune cells (https://cistrome.shinyapps.io/timer/). The TIMER database includes 10,897 samples across 32 cancer types from TCGA. It was used to study the relationship between SCNA and six tumor-infiltrating immune cell subsets (B cells, CD4 T cells, CD8 T cells, macrophages, neutrophils, and DC) in LUAD.

### Immunohistochemistry

Immunohistochemistry (IHC) was described in our published study [14]. Undergoing deparaffinized, rehydrated, and antigen retrieval (EDTA 9.0), slides were blocked with 3% hydrogen peroxide and 5% goat serum, then being incubated overnight at 4 °C with primary antibodies. In the next day, slides were incubated with enhancer and secondary antibody at room temperature. A DAB Substrate Kit was used for chromogenic reaction. Finally, the sections were stained with hematoxylin, then dehydrated, cleared, and evaluated. Primary antibodies include anti-CD4 antibody (ab133616, Abcam, USA, 1:100), anti-CD8 antibody (SP16, Thermo Fisher Scientific, USA, 1:100), anti-CD68 antibody (KP1, Thermo Fisher Scientific, USA, 1:200), and anti-CD11c antibody (EP1347Y, Abcam, USA, 1:200). Immunostaining was evaluated under light microscopy at 400× magnification by two independent pathologists. The absolute number of immune cells was counted manually. The total number of stained immune cells (in the central tumor and peritumoral stroma) was included in the analyses.

### Statistical analysis

Survival curves were constructed using the Kaplan-Meier method, and differences in the progression-free survival (PFS) and overall survival (OS) among groups were assessed by the log-rank test. The association between SCNA and clinicopathological parameters was estimated by the Fisher exact test. Cox proportional models were used for univariate and multivariable analyses. All statistical analyses were conducted with R software (version 3.5.3).

## Result

### SCNA predicted survival of NSCLC patients in the CIK + CT cohort

**Fig. 1:**
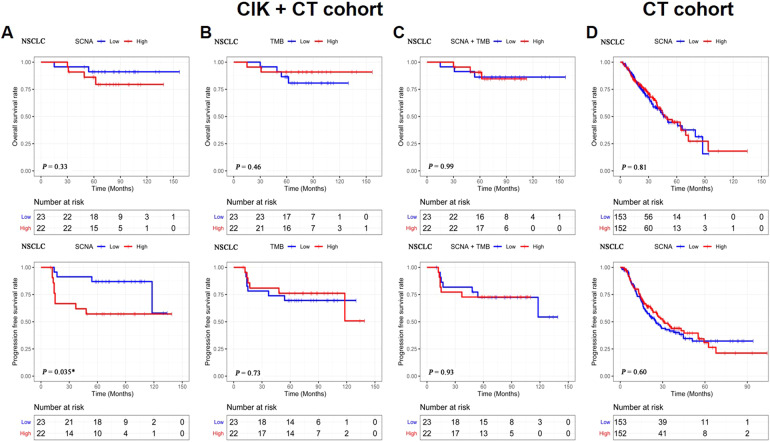
Survival analysis for SCNA or TMB of NSCLC patients in CIK + CT cohort and CT cohort. **A** OS and PFS of SCNA in NSCLC patients (CIK + CT cohort, *n* = 45, median SCNA: 1788). **B** OS and PFS of TMB in NSCLC patients (CIK + CT cohort, median TMB: 4.26). **C** OS and PFS of SCNA + TMB in NSCLC patients (CIK + CT cohort). **D** OS and PFS of SCNA in NSCLC patients (CT cohort, *n* = 305, median SCNA: 153). The Kaplan-Meier method was used to compare the survival rates, which were analyzed with the log-rank test. Significance is given as *(*P* < 0.05), **(*P* < 0.01), and ***(*P* < 0.001).

Tumor samples from 45 patients with different types of NSCLC, including LUAD (60%) and LUSC (40%) were profiled by WES (Table 1). We calculated SCNA and TMB and tested their association with the survival of NSCLC patients receiving CIK + CT. Firstly, we grouped patients into low and high SCNA groups based on the median and did the same for TMB. We found that SCNA, TMB, and SCNA + TMB were not related to OS (*P* = 0.33, *P* = 0.46, *P* = 0.99, respectively; Fig. 1A−C). However, SCNA was significantly associated with PFS (*P* = 0.035), while TMB and SCNA + TMB were not (*P* = 0.73, *P* = 0.93, respectively). Furthermore, we used a control group with 305 NSCLC patients from TCGA who received CT to compare the prognostic value of SCNA, and found no correlation between SCNA and survival (Fig. 1D).

### SCNA predicted survival of LUAD patients in the CIK + CT cohort

**Fig. 2:**
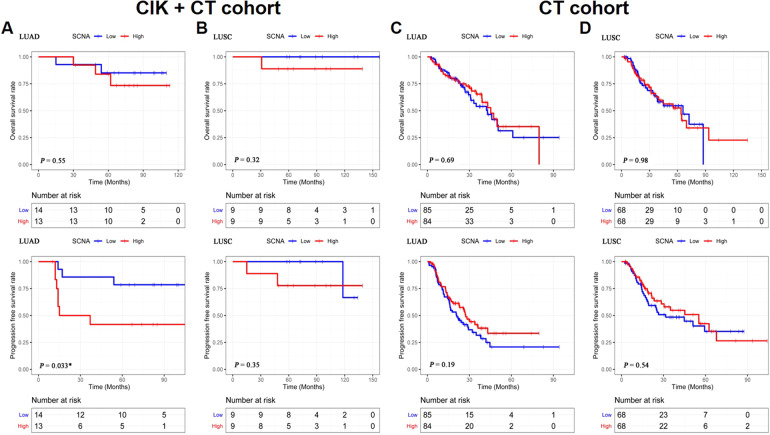
Survival analysis of LUAD and LUSC patients in CIK + CT cohort and CT cohort based on SCNA. **A** OS and PFS of SCNA in LUAD patients (CIK + CT cohort, *n* = 27, median SCNA: 1848). **B** OS and PFS of SCNA in LUSC patients (CIK + CT cohort, *n* = 18, median SCNA: 1519). **C** OS and PFS of SCNA in LUAD patients (CT cohort, *n* = 169, median SCNA: 141). **D** OS and PFS of SCNA in LUSC patients (CT cohort, *n* = 136, median SCNA: 172). Significance is given as *(*P* < 0.05), **(*P* < 0.01), and ***(*P* < 0.001).

A recent study reported that genetic alterations may differ between LUAD and LUSC [15]. We assessed whether SCNA was associated with response to CIK + CT treatment among different NSCLC subtypes. First, we analyzed the OS and PFS in LUAD and LUSC patients, respectively. In the CIK + CT cohort, LUAD patients with SCNA below the median had a longer PFS than patients with SCNA above the median (*P* = 0.033; Fig. 2A). There were no differences in the OS and PFS between LUSC patients with low and high SCNA (*P* = 0.32, *P* = 0.35, respectively; Fig. 2B). In contrast, survival outcomes among LUAD or LUSC patients from TCGA who received CT did not correlate with SCNA (Fig. 2C, D), suggesting that SCNA level cannot distinguish patients who may show better clinical response with chemotherapy.

For determining the relationship between the SCNA level and patient’s clinical parameters, we performed a Fisher test analysis to compare the low and high SCNA groups of LUAD patients, which revealed that SCNA was not related to gender, age, smoking, TMB, and tumor stage (Table 2). Similar results were observed in LUSC patients (Table 2). Collectively, SCNA may be a better predictive indicator to identify patients who are most likely to benefit from CIK + CT treatment.

**Table 2:** Relationship Between SCNA and Clinical Data.

Variables	SCNA-LUAD		*P* value	SCNA-LUSC		*P* value
	Low	High		Low	High	
Gender, *n*						
Male	10	8	0.6946	7	9	0.4706
Female	4	5		2	0	
Age, y, *n*						
<60	7	8	0.7036	5	5	1.0000
≥60	7	5		4	4	
Smoking, *n*						
No	5	10	0.0542	4	1	0.2941
Yes	9	3		5	8	
Tumor stage, *n*						
І	6	6	0.6835	5	5	1.0000
II	5	2		2	2	
III	2	4		2	1	
IV	1	1		0	1	
TMB						
Low, *n*	6	8	0.4495	7	2	0.0567
High, *n*	8	5		2	7	
Progression, *n*						
No	11	6	0.1201	8	7	1.0000
Yes	3	7		1	2	
Survival, *n*						
No	2	3	0.6483	0	1	1.0000
Yes	12	10		9	8	

### Development of prediction models with SCNA in LUAD

We found that SCNA was significantly associated with PFS in LUAD patients but not in LUSC patients. Therefore, we excluded LUSC from subsequent analyses. We utilized the Cox proportional hazards regression model to analyze whether SCNA can predict survival in LUAD patients (Table 3). On univariate analysis, the SCNA level negatively correlated with the PFS (hazard ratio [HR] = 3.9559, 95% confidence interval [CI]: 1.0161−15.502, *P* = 0.0474). On multivariate analysis, only SCNA remained statistically significant (HR = 13.187, 95% CI: 1.6832−103.313, *P* = 0.0141).

**Table 3:** Progression-Free Survival: CIK Cohort.

Characteristic	HR (95% CI)	*P* value
**Univariate analysis**		
Gender (Male vs. Female)	0.7542 (0.1948−2.9201)	0.683
Age (<60 vs. ≥60)	0.6801 (0.1915−2.4159)	0.5511
SCNA (Low vs. High)	3.9559 (1.0161−15.502)	0.0474*
TMB (Low vs. High)	2.1424 (0.6038−6.6014)	0.2383
Smoking (No vs. Yes)	0.8424 (0.2375−2.9878)	0.7907
TNM (I vs. II, III, IV)	1.6214 (0.8802−2.9869)	0.121
**Multivariate analysis**		
Gender (Male vs. Female)	1.5182 (0.2778−8.296)	0.6299
Age (<60 vs. ≥60)	0.5637 (0.064−4.964)	0.6055
SCNA (Low vs. High)	13.187 (1.6832−103.313)	0.0141*
TMB (Low vs. High)	3.6576 (0.6155−21.737)	0.1538
Smoking (No vs. Yes)	4.9309 (0.384−63.324)	0.2206
TNM (I vs. II, III, IV)	1.4796 (0.7019−3.119)	0.3032

Bold values indicate statistical significance *P* < 0.05.

**Fig. 3:**
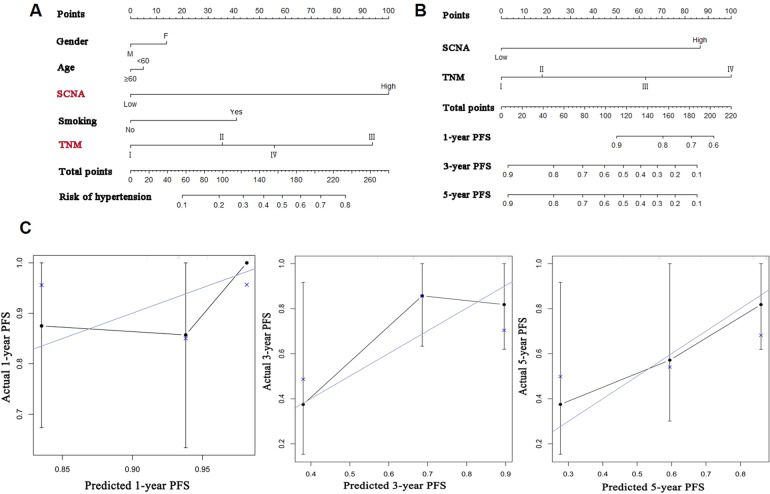
Nomogram for predicting PFS of LUAD patients in CIK + CT cohort. **A** Predictive nomogram of gender, age, SCNA, smoking and TNM. The nomogram is used to adding up the total points measured by the points scale for each variable. **B** Nomogram showed the assessment of PFS with SCNA and TNM. **C** The calibration curve for predicting PFS at 1-year, 3-year and 5-year.

Multivariable clinicopathological characteristics were used as prognostic factors for building a nomogram to predict PFS in LUAD patients. Among the five characteristics, SCNA and TNM were significantly associated with PFS (Fig. 3A) and were selected as variables to develop the nomogram (Fig. 3B). The C-index for PFS prediction was 0.8. Additionally, the 1-, 3- and 5-year PFS probability could be calculated with this nomogram. For example, a patient with stage III cancer with high SCNA will have a total risk score of 150 points, which corresponds to 1-, 3-, and 5-year survival probability of 81%, 30%, and 28%, respectively. Figure 3C shows the calibration plots for the probability of survival at 1-, 3-, and 5-year after CIK + CT treatment, displaying an optimal agreement between the prediction by nomogram and actual observation at 3- and 5- year. The findings support the prognostic potential of SCNA in LUAD patients.

### Association between SCNA with immune cell infiltration

**Fig. 4:**
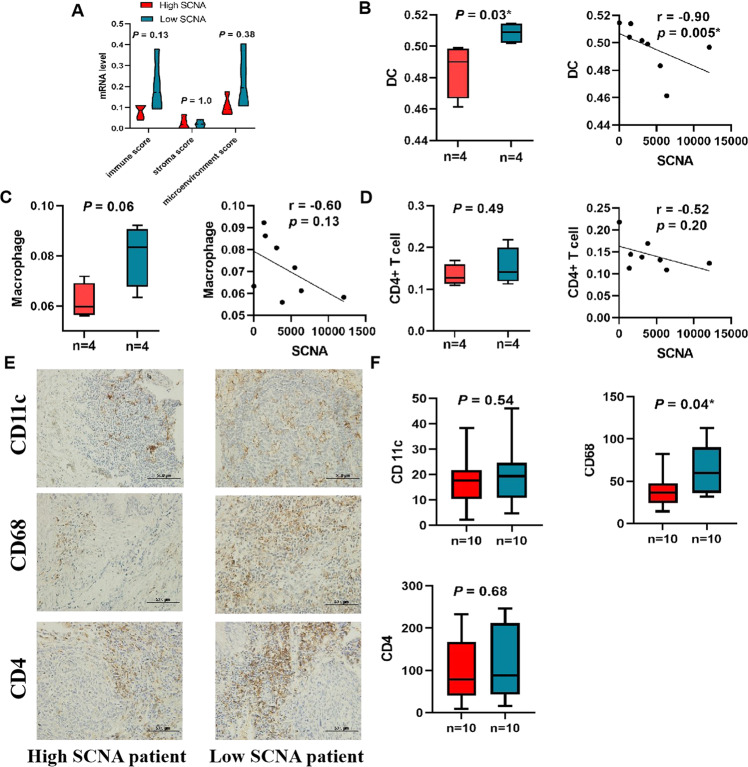
Correlations between SCNA and immune cells in LUAD patients in CIK + CT cohort. **A** Correlations between SCNA, immune score, stroma score and microenvironment score. **B**−**D** Correlations between SCNA and the infiltration levels of three immune cells (DC, Macrophage and CD4 + T cell) from RNA sequencing. **E**, **F** Immunohistochemical analysis of DC, Macrophage and CD4 + T cell. Significance is given as *(*P* < 0.05), **(*P* < 0.01), and ***(*P* < 0.001).

After defining the prognostic value of SCNA, we explored the association between SCNA and tumor-infiltrating immune cells in eight LUAD patients based on RNA sequencing. First, based on the median, we classified eight patients into low and high SCNA groups and assessed the immune microenvironment by xCell to explore the association of SCNA level with the immune score, stroma score, and microenvironment score (Fig. 4A). Immune score (*P* = 0.13) and microenvironment score (*P* = 0.38) were higher in the low SCNA group than in the high SCNA group. The trend gave us a clue to the underlying association between SCNA and immune cell infiltration, and that benefits of CIK + CT may be linked with the immune microenvironment. We further assessed the composition of the tumor immune microenvironment by the TIMER algorithm and found that patients with low SCNA exhibited higher infiltration of DC (*P* = 0.03) (*P* = 0.06, Fig. 4B, C), however, there were no statistically significant differences in macrophages, CD4 + T cells, B cells, CD8 + T cells, and neutrophils between the high and low SCNA groups (Fig. 4C, D, Supplementary Figure 1A−D). Limited by the number of patients sequenced, these correlations need further exploration.

### The SCNA of genes and their association with CIK + CT response

To explore the underlying molecular mechanisms that affect the CIK + CT response. We analyzed copy number profiles of 17 progression and 10 non-progression patients. The progression of tumors often involves amplifications and deletions of genomic DNA. Compared to non-progression group, the top 10 most significant amplification genes included ADK, AMIGO2, ASIC1(ACCN2), COL2A1, FAM72D, OLFML3, PFN1P2 and PPIAL4G in progression group (Supplementary Fig. 2A). Among these genes, high expression of AMIGO2 and ASIC1 has been reported to promote tumor metastasis. In non-progression group, SCNA genes represented deletion status. CACNA2D4, LRTM2 and MLF2 genes were significant deletion (Supplementary Figure 2B). These results provide us new knowledge by illuminating modes of genomic alteration that overexpression of oncogenes promoting chromosome instability has been resulted in increased aggressiveness and poor prognosis.

## Discussion

SCNA is a hallmark of cancer and is also reported to have a predictive role in cancer, with high SCNA showing a correlation with poorer prognosis in the majority of the patients. For lung cancer detection, SCNA has the potential to improve the sensitivity of cancer screening; [16] regarding the prognostic value of SCNA in lung cancer, patients with low SCNA showed a positive response to immune checkpoint inhibitors (ICI) [11]. Our work unfolded in the following course. First, we compared the prognostic ability of SCNA, TMB and SCNA + TMB in NSCLC patients treated with CIK + CT. Second, we identified SCNA as a survival factor to carry out survival and Cox regression analysis in LUAD patients treated with CIK + CT. Third, we proposed a practical nomogram model to make a precisive prediction.

CIK cell with CD3 + CD56 + phenotype, which is also called the natural killer T cells, have mainly MHC-unrestricted antitumor activity against cancer. And an increasing number of clinical trials have indicated that CIK treatment has high efficacy on clinical responses. Some studies have reported that CIK + CT for cancer therapy showed better efficacy than chemotherapy alone, and prolonged patient survival and reduces tumor progression [17, 18]. In our study, we calculated SCNA and TMB and tested their association with the survival of NSCLC patients receiving CIK + CT. The result revealed a significant association between SCNA with PFS in NSCLC patients treated with CIK + CT as compared to TMB and SCNA + TMB. However, SCNA could not predict the survival of CT. Interestingly, SCNA was significantly associated with the survival of LUAD but not with LUSC patients. These two NSCLC subtypes have both unique and shared clinical and histopathological characteristics. In addition, LUAD harbors high rates of genomic rearrangement and somatic mutation, and these molecular features are associated with the clinical, pathological, and prognostic features of LUAD.

LUAD patients with low SCNA treated with CIK + CT demonstrated long-term survival, while SCNA was not associated with prognosis in patients receiving CT. CIK cells have been identified as potential tools for adoptive T cell immunotherapy for providing anti-tumoral activity in cancer patients [19, 20]. The predictive potential of SCNA in immunotherapy may be explained by the association between SCNA and the immune microenvironment. Some studies have supported an inverse association between SCNA and immune cell infiltration [8, 21]. A study classified triple-negative breast cancer (TNBC) patients with survival information from TCGA into good and poor prognosis groups [21]. The good prognosis TNBC samples, defined as the immune-rich subtype based on immune infiltration, harbored fewer SCNA. Pan-cancer TCGA analysis revealed that most of the lowly expressed genes in the high SCNA group were associated with the immune system, especially cytotoxic immune cells [8]. Cancers with low immune infiltration may be associated with an immune escape that allows tumor evolution towards greater genomic diversity. CD3 + CD56 + CIK cells with potent cytotoxicity display wide MHC-unrestricted antitumor activity and influence adoptive cellular immunotherapy [22]. SCNA and CIK cells were intensely associated with the immune system, which may explain why SCNA can predict the efficacy of CIK + CT but not CT alone. Combining SCNA and the practical need of clinical work, we have chosen SCNA and TNM for a nomogram. The result exhibited an optimal agreement between the prediction by nomogram and actual observation in 3- and 5- year. The prognostic study of LUAD will be of great help to physicians.

We also evaluated the protein levels of DC, macrophages, CD4 + T cells, and CD8 + T cells in the CIK + CT cohort, and the results were consistent with the TIMER analysis that SCNA was inversely associated with the expression levels of DC and macrophages. A study reported that low SCNA levels correlated with low glucose consumption, decreased immune evasion, and contributed to immune cell recognition [23]. Patients with intense fluorodeoxyglucose uptake had significantly decreased mast cells, neutrophils, and monocytes. DCs perform specialized antigen-processing and activate either CD4 + or CD8 + T cells, which mediate the killing of cancer cells and are a positive prognostic factor in multiple solid tumors [24]. As we reported earlier, SCNA may have a role in the antitumor immune response, in addition to affecting a variety of gene products related to the immune system [25].

Additionally, we uncovered relationships between prognosis and SCNA of specific genes. SCNA plays critical roles in activating oncogenes and inactivating tumor suppressors [26]. When comparing the two groups with or without progression, we found that progression group showed more oncogenes amplification status, however, non-progression group presented more oncogenes deletion status. In progression group, high expression of AMIGO2 and ASIC1 has been reported to promote tumor metastasis [27, 28]. Therefore, copy number amplification leads to overexpression of these genes, which further leads to tumor development. And in non-progression group, copy number deletion contributing to low expression of MLF2 has been reported to inhibit tumor metastasis [29]. However, other statistically different genes were not found to be associated with tumor prognosis, which needed to be studied further.

In summary, our study suggests that high SCNA is linked with poor PFS in LUAD patients receiving CIK + CT treatment, which may facilitate therapeutic interventions that could improve the efficacy of the current combination of immunotherapy and chemotherapy. However, due to the small sample size, validation of our findings in a larger cohort of patients is needed.

## Supplementary information

Supplementary figure

Supplementary table 1

Supplementary table 2

## References

[1] Herbst R, Morgensztern D, Boshoff CJN (2018). The biology and management of non-small cell lung cancer. Nature.

[2] Ettinger DS, Wood DE, Aisner DL, Akerley W, Bauman J, Chirieac LR (2017). Non-Small Cell Lung Cancer, Version 5.2017, NCCN Clinical Practice Guidelines in Oncology. J Natl Compr Cancer Netw: JNCCN.

[3] Linn Y, Hui KJL (2003). Cytokine-induced killer cells: NK-like T cells with cytotolytic specificity against leukemia. Lymphoma.

[4] Yang L, Du C, Wu L, Yu J, An X, Yu W (2017). Cytokine-induced killer cells modulates resistance to cisplatin in the A549/DDP cell line. J Cancer.

[5] Huang J, Kan Q, Lan, Zhao X, Zhang Z, Yang S (2017). Chemotherapy in combination with cytokine-induced killer cell transfusion: An effective therapeutic option for patients with extensive stage small cell lung cancer. Int Immunopharmacol.

[6] Yang L, Ren B, Li H, Yu J, Cao S, Hao X (2013). Enhanced antitumor effects of DC-activated CIKs to chemotherapy treatment in a single cohort of advanced non-small-cell lung cancer patients. Cancer Immunol Immunother.

[7] Holland A, Cleveland D (2009). Boveri revisited: Chromosomal instability, aneuploidy and tumorigenesis. Nat Rev Mol Cell Biol.

[8] Davoli T, Uno H, Wooten E, Elledge SJS (2017). Tumor aneuploidy correlates with markers of immune evasion and with reduced response to immunotherapy. Science.

[9] Chalmers ZR, Connelly CF, Fabrizio D, Gay L, Ali SM, Ennis R (2017). Analysis of 100,000 human cancer genomes reveals the landscape of tumor mutational burden. Genome Med.

[10] Marabelle A, Fakih M, Lopez J, Shah M, Shapira-Frommer R, Nakagawa K (2020). Association of tumour mutational burden with outcomes in patients with advanced solid tumours treated with pembrolizumab: Prospective biomarker analysis of the multicohort, open-label, phase 2 KEYNOTE-158 study. Lancet Oncol.

[11] Kim HS, Cha H, Kim J, Park WY, Choi YL, Sun JM (2019). Genomic scoring to determine clinical benefit of immunotherapy by targeted sequencing. Eur J Cancer (Oxf, Engl: 1990).

[12] Kou F, Wu L, Guo Y, Zhang B, Li B, Huang Z (2021). Somatic copy number alterations are predictive of progression-free survival in patients with lung adenocarcinoma undergoing radiotherapy. Cancer Biol Med.

[13] Li R, Wang C, Liu L, Du C, Cao S, Yu J (2012). Autologous cytokine-induced killer cell immunotherapy in lung cancer: A phase II clinical study. Cancer Immunol Immunother.

[14] Kou F, Sun H, Wu L, Li B, Zhang B, Wang X (2020). TOP2A promotes lung adenocarcinoma cells’ malignant progression and predicts poor prognosis in lung adenocarcinoma. J Cancer.

[15] Zhang XC, Wang J, Shao GG, Wang Q, Qu X, Wang B (2019). Comprehensive genomic and immunological characterization of Chinese non-small cell lung cancer patients. Nat Commun.

[16] Danielsen H, Pradhan M, Novelli M (2016). Revisiting tumour aneuploidy - the place of ploidy assessment in the molecular era. Nat Rev Clin Oncol..

[17] Zhou ZQ, Zhao JJ, Pan QZ, Chen CL, Liu Y, Tang Y (2019). PD-L1 expression is a predictive biomarker for CIK cell-based immunotherapy in postoperative patients with breast cancer. J Immunother Cancer.

[18] Gao X, Mi Y, Guo N, Xu H, Xu L, Gou X (2017). Cytokine-induced killer cells as pharmacological tools for cancer immunotherapy. Front Immunol..

[19] Introna M (2017). CIK as therapeutic agents against tumors. J Autoimmun..

[20] Introna M, Correnti F (2018). Innovative Clinical Perspectives for CIK Cells in Cancer Patients. Int J Mol Sci.

[21] Karn T, Jiang T, Hatzis C, Sänger N, El-Balat A, Rody A (2017). Association between genomic metrics and immune infiltration in triple-negative breast cancer. JAMA Oncol.

[22] Wang X, Yu W, Li H, Yu J, Zhang X, Ren X (2014). Can the dual-functional capability of CIK cells be used to improve antitumor effects?. Cell Immunol.

[23] Han S, Oh J, Kim J (2021). Immune microenvironment of the gene signature reflecting the standardised uptake value on F-fluorodeoxyglucose positron emission tomography/computed tomography in head and neck squamous cell carcinoma. Ann Nucl Med..

[24] Saxena M, Bhardwaj N (2018). Re-emergence of dendritic cell vaccines for cancer treatment. Trends Cancer.

[25] Kou F, Wu L, Ren X, Yang L (2020). Chromosome abnormalities: New insights into their clinical significance in cancer. Mol Ther Oncolytics.

[26] Zack TI, Schumacher SE, Carter SL, Cherniack AD, Saksena G, Tabak B (2013). Pan-cancer patterns of somatic copy number alteration. Nat Genet.

[27] Kanda Y, Osaki M, Onuma K, Sonoda A, Kobayashi M, Hamada J (2017). Amigo2-upregulation in tumour cells facilitates their attachment to liver endothelial cells resulting in liver metastases. Sci Rep..

[28] Gupta SC, Singh R, Asters M, Liu J, Zhang X, Pabbidi MR (2016). Regulation of breast tumorigenesis through acid sensors. Oncogene.

[29] Yang J, Cao D, Zhang Y, Ou R, Yin Z, Liu Y (2020). The role of phosphorylation of MLF2 at serine 24 in BCR-ABL leukemogenesis. Cancer Gene Ther.

