# Supplementary material for: Somatic copy number alteration predicts clinical benefit of lung adenocarcinoma patients treated with cytokine-induced killer plus chemotherapy
Source: Cancer Gene Ther. 2022 Jan 12;29(8-9):1153–9. doi: 10.1038/s41417-021-00422-5 (PMC9395268; doi:10.1038/s41417-021-00422-5)

Supplementary Figure 1. Correlations between SCNA B cell, CD8+ T cell and neutrophil in LUAD patients in CIK+CT cohort. (A-C) The distribution of B cell, CD8+ T cell and neutrophil in high and low SCNA from RNA sequencing. (D) Immunohistochemical analysis of CD8+T cell expression in LUAD patients.


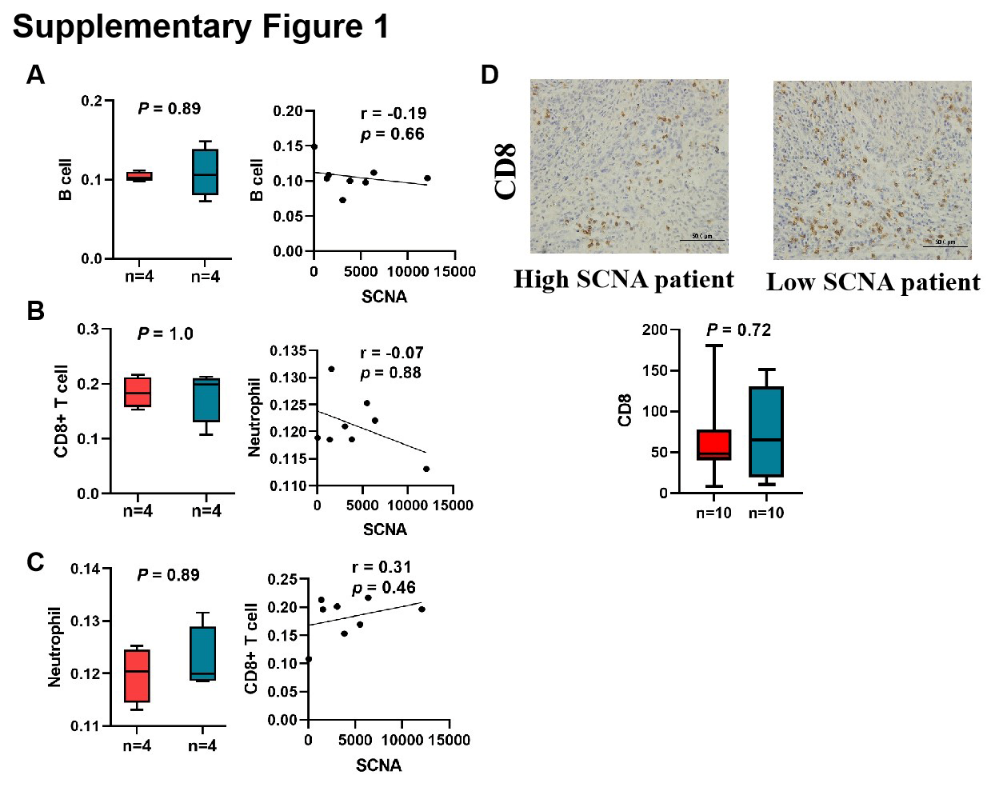


Supplementary Figure 2. The copy number amplification and deletion genes. (A) Differential amplification genes between progression group and non-progression group in LUAD patients in CIK+CT cohort. (B) Differential deletion genes between progression group and non-progression group in LUAD patients in CIK+CT cohort.


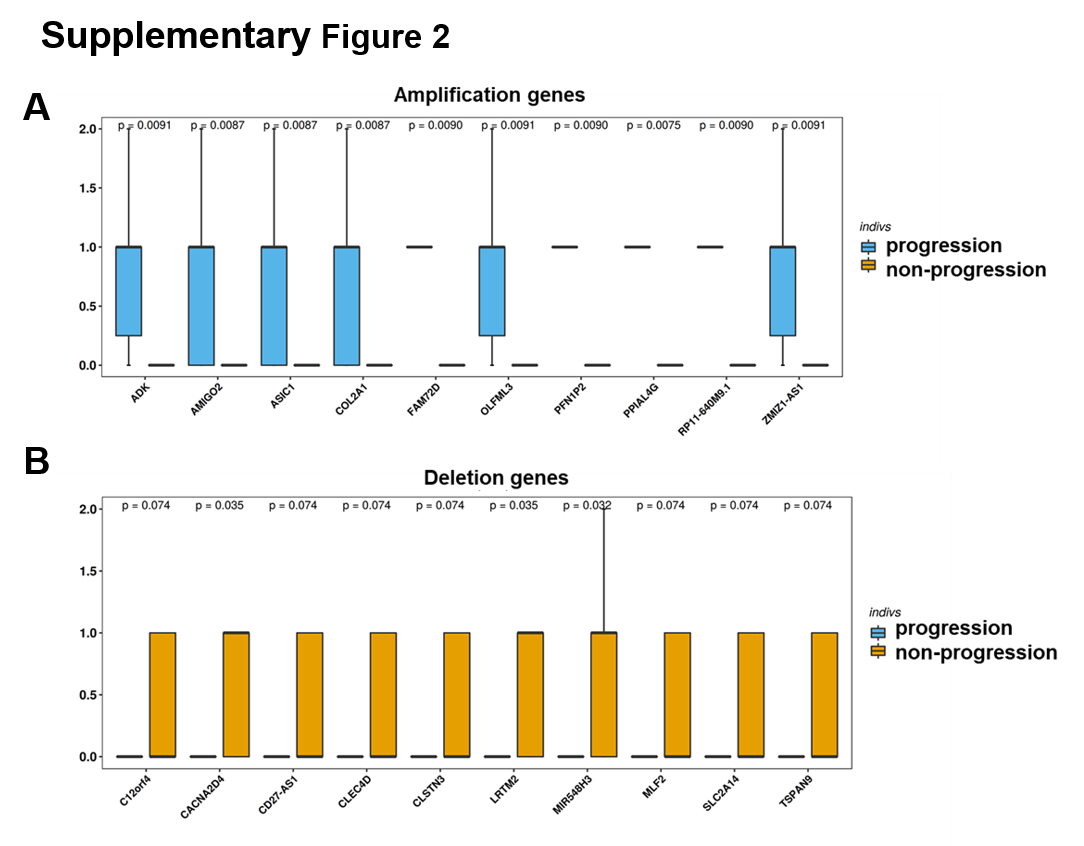

Supplement: Supplementary file 1 — Supplementary figure [file 41417_2021_422_MOESM1_ESM.doc]
